# Examining smoking and cessation during pregnancy among an Appalachian sample: a preliminary view

**DOI:** 10.1186/1747-597X-2-14

**Published:** 2007-05-07

**Authors:** Lesley Cottrell, Mark Gibson, Carole Harris, Alia Rai, Sabera Sobhan, Traci Berry, Bonita Stanton

**Affiliations:** 1 PO Box 9214 Robert C. Byrd Health Sciences Center. Department of Pediatrics, West Virginia University School of Medicine, Morgantown, WV 26506-9214, USA; 2 Department of Obstetrics and Gynecology, University of Utah Health Sciences Center, Salt Lake City, Utah 84132-2209, USA; 3 P.O. Box 9100 Robert C. Byrd Health Sciences Center. Health Research Center, West Virginia University School of Medicine, Morgantown, WV 26506-9214, USA; 4 Department of Counseling Psychology, One Hermann Museum Circle Drive, Houston, TX 77004, USA; 5 P.O. Box 6122. Department of Counseling Psychology, West Virginia University. Morgantown, WV, USA; 6 3901 Beaubian, 1T110 Children's Hospital of Michigan. Wayne State University School of Medicine. Detroit, MI 48201, USA

## Abstract

**Background:**

Several transitions that a woman experiences prenatally may influence her desire or ability to discontinue smoking. This study explores the role of smoking for young, Appalachian, nulliparous pregnant women and their plans for smoking during their pregnancies.

**Results:**

The reports of women and their male partners were taken from baseline interviews conducted during the first trimester of pregnancy. Cigarette smoking appeared to be more than an isolated addictive activity; rather, smoking was interwoven in women's social and personal realms, often changing as their perceptions of self changed. Women and their partners who continued to smoke appeared to be depressed, reject authority, and perceived little control over issues related to being pregnant.

**Conclusion:**

These findings support the argument that standard substance use treatments and polices based on stages-of-change theories may not be effective for all individuals particularly those experiencing significant developmental changes in their lives. Greater success might be obtained from treatment programs designed to recognize the impact of these transitions as it relates to the substance use. The changing experiences of pregnant women in terms of their identity development, views of others, and their relationships have not been adequately addressed in existing cessation programs. Empirically-based interventions targeting these lifestyle characteristics may lead to increased cessation success among pregnant women.

## Introduction

Recent reports of smoking during women's pregnancies indicated that 11.4% of all women giving birth in the United States continued to smoke [1]. This rate had declined from 18.4% in 1990 [1]. State declines in maternal smoking behaviors have been variable. According to 1998 West Virginia (WV) vital statistics, 25% of pregnant women smoked during their pregnancies, and women of childbearing age (18–34 years) smoked at a rate of about 35% [2], this rate had only declined by 5.8% since 1998.

There is no dearth of literature regarding the general topic of smoking and smoking cessation among pregnant women [3-10]. These studies have provided detailed descriptions of smoking and smoking cessation rates, trends in these rates, and variations in smoking by race, parity, geographic location and other demographic variables. Finally, quantitative and qualitative research has explored factors associated with smoking throughout pregnancy and those associated with smoking cessation [4,6,9,11].

A wide range of behavioral approaches towards smoking cessation have been proffered; a recurring theme among many of the behavioral interventions most frequently cited as "effective", (i.e., "Smoking Cessation or Reduction in Pregnancy Trial" by Windsor and colleagues, is their explicit or implicit grounding in Prochaska's, DiClemente's, and Norcross' "Stage of Change" theory [12-14].

Herein lies a paradox; while there is a vast literature on the topic of prenatal smoking and an intervention theme invoking stages related to one's readiness to change, there has been but minimal exploration in the literature of the changes inherent in pregnancy – particularly the first pregnancy – and how these changes may impact a woman's willingness, desire, and/or ability to discontinue smoking. That is, to date the issue has been largely explored from a behavioral or functional perspective, rather than a dynamic one.

The importance of understanding the relationship between an individual's developmental stage and his or her behavior or behavioral response to an intervention has been a cornerstone of research among children and adolescents for at least the past decade [15,16]. This same theme has not predominated in the adult behavioral literature, although certainly transitions from one developmental stage to another occur throughout one's life. Regardless of whether a developmental approach is required for behavioral research among adults, a compelling argument can be made for it to be considered in understanding women as they transition towards motherhood, especially for the first time.

The transition to motherhood is accompanied by significant biological, social, and psychological events [17-20]. The transition has been described as an opportunity (or a necessity) for a woman to develop "new possible selves" [21] referring to the significance of the maternal role in the woman's life and a new sense of herself. Researchers have argued that pregnancy provides an opportunity for women to describe for themselves, through experimentation, their future role as a mother [21]. Thus, during pregnancy a woman can grow, regress [22], or perhaps enter a dynamic state of intra-personal change marked by regression and growth [19]. The significance of this state of intra-personal flux is that the application of a "stage of change" model to behavioral change may be less robust in application to pregnant women than when applied to individuals during a more static or stable period of development --- as perhaps mid-adulthood might be. Alternatively, the dynamic nature of pregnancy may open the opportunity for a fluid and rapid progression through the stages of change.

Arguably, several of the transitions that a woman experiences prenatally may influence her desire or ability to discontinue smoking as a substantial literature documents the importance of social influences and intrinsic psychologic attributes of the woman on smoking initiation and cessation. For example, during pregnancy a woman may assume a more private, less public world [19]. Thus, someone who derived great pleasure from large social gatherings in which smoking was a shared activity may no longer find herself attracted to such settings. She may turn towards family (i.e., her partner or her mother, father or siblings) or sometimes another pregnant woman [23]. The relative acceptance of her smoking by these members may assume greater or lesser importance in her life. These transitions in focus can be in flux; often with some return to the partner in mid-pregnancy [24] and thus a perception of "readiness to change" or willingness to quit smoking may also be in flux.

Therefore, we undertook the present study to explore conceptualizations of pregnancy, relationships and smoking might serve as the foundation for a dynamic approach to smoking cessation among young pregnant women or that would affirm the use of the stages of change theory for smoking cessation efforts during pregnancy. Our exploration was predicated on the assumption that a woman's first pregnancy marks a revolutionary change in her self-identity and her relationship with, and dependence on, those around her. These changes may be especially remarkable for the adolescent and young adult mother-to-be whose self-identity is still undergoing definition, whose actions may be guided in part by rebellion against authority figures in her life, and whose concept of intimacy may already be characterized more as dependency than as mutuality [25,26]. Therefore, for the pregnant adolescent or young adult, smoking may well be not simply a habit or addictive behavior but an image-defining activity [27].

Accordingly, in this study, we sought to determine the role of smoking in a young (i.e., 17 to 25-year-old), nulliparous pregnant woman's sphere of self and her social interactions. In this paper we report on the results from the baseline interviews [pregnant women and their significant others] which occurred during the first trimester of pregnancy and address the role of smoking in women's daily life and interactions within the domestic, friendship, and work sphere's as the women progress through the first trimester of their pregnancies.

## Methods

### Subjects

Women ages 17 to 25 attending a prenatal clinic within a university-based medical teaching college and tertiary health care screening center [Physician's Office Center – POC] were invited to participate in this study. The POC is a large, multi-group practice providing patients with access to primary and specialty care services. Women were eligible to participate if they had not had a previous pregnancy resulting in a live birth and if they smoked cigarettes at least up until the time that they learned of their pregnancy. Women who had stopped smoking upon learning of their pregnancies were enrolled in this study in order to compare potential differences in the factors that led them to change their smoking behavior with women who continued to smoke after hearing of their pregnancies.

Women's male partners were also invited to participate in separate interviews regardless of their smoking behavior if they were available during the initial recruitment visit with the eligible woman. Open-ended interviews and seven measures assessing demographic, psychological and social characteristics of the mother were administered to the women. The same procedures were offered to, and implemented for the women's significant others or partners if present.

### Procedures

In an attempt to obtain women's (and their partner's) initial responses (smoking and societal views) to their pregnancies, we emphasized recruitment of women who were in their first trimester; however, women who were further along in their pregnancies were also eligible to participate in this study. Obstetric members of our team identified women based on their eligibility status who were coming in for a routine clinic visit in the tertiary care center. Therefore, a convenience sample of women and their partners was used in this study.

Following their medical visit, a research staff member approached women and their partners after their parity and smoking status had been determined by the nursing staff. After a complete description of the study procedures had been provided and written, informed consent was obtained, separate open-ended interviews were administered to each woman and her partner (when available). The interviews, each lasting approximately 60 minutes were audiotaped. Subsequently, standardized, quantitative measures were self-administered (paper and pencil) by each participant in a separate clinic room. Because we were interested in exploring new ways of conceptualizing the relationship of smoking and pregnancy, we did not specifically hypothesize relationships between perceptions of smoking and self with intentions and actions relevant to smoking. Rather, using grounded theory [28] to organize our study approach, we allowed women to tell us their own stories about themselves, their relationships with their partners, their feelings about smoking and other behaviors that they chose to discuss. The use of both qualitative and quantitative (including some standardized questionnaires) permits an interdisciplinary approach that may not be possible through the use of a single data-gathering methodology. Human behavior is sufficiently complex that researchers are increasingly recognizing the need to utilize more than a single data-gathering methodology [29]. Study team members were available to answer questions about the questionnaire or to read questionnaire items to participants when needed. Questionnaires took approximately 30 minutes to complete. The Institutional Review Board at West Virginia University approved the study.

### Measures

#### Structured individual interviews

Following brief introductory remarks and questions regarding the woman's pregnancy status and general smoking history (e.g., why and when did she start), an individual interview guide explored two main domains: (1) the triggers and consequences (physiological, social, and emotional) of smoking (and smoking cessation) and (2) potential changes in the triggers and consequences of smoking before and after becoming pregnant. Women's partners were asked questions related to their own smoking behavior, their perceptions regarding the women's smoking behaviors, their perceived outlooks of the women's future cigarette use. Additional queries were made about the woman's (and partner's) descriptions of the duration and nature of the couple's relationship and how it changed after their discovery of the pregnancy. The role of smoking in the woman's (and partner's where appropriate) life before and after becoming pregnant was woven into this examination of the relationship over time.

#### Standardized, quantitative measures

a) Mullens' five-choice *Multiple Choice Smoking *History [30] was used to measure the current smoking status of the women and their partners. Such items as "Which of the following statements best describes your cigarette smoking" were connected with multiple-choice responses to reduce fabrication compared to a dichotomous measure.

b) Albrecht's 7-item modification of the *Fagerstrom Nicotine Tolerance Scale *(FNTS) [31] is specifically designed for adolescents and young adults. Scores range from 1 to 11. High levels of dependence are correlated with scores over 6.

c) Beck's 21-item *Beck Depression Inventory-II *[32] to measure depressive symptoms experienced by men and women. Each item is scaled 0 to 3; a score of 0–13 indicates minimal depressive symptoms, of 14 to 19 mild depressive symptoms, of 20 to 28 moderate depressive symptoms, and above 29, severe depressive symptoms.

d) Couch's five-item *Naysaying Low F Scale *[33] uses a Likert-scale format to assess authoritarianism-rebellion. Response choices range from 1 (strongly disagree) to 7 (strongly agree). The range is from 5 (High F) to 35 (Low F). Higher scores indicate a rejection of authority. Psychometric properties are not documented. At the end of this measure we asked six questions assessing maternal perceptions of her partner's smoking now and before her pregnancy, maternal perceptions of her partner's views on her smoking (now and before her pregnancy), and maternal perception's of her partner's views on his own smoking now and before her pregnancy. (These questions are partner reversed when asked of males).

#### Thematic analysis

Audiotapes were transcribed and then entered into ETHNOGRAPH [34], software developed for managing large-text data sets. Each transcript of an interview was initially reviewed and coded in an open-ended fashion by two coders. Subsequently we engaged in axial coding to determine where there was overlap between the concepts identified by the two coders of each of the transcripts and to further refine the constructs identified. This process was repeated to generate a dictionary of code terms until we had reached data saturation – that is no new ideas were emerging despite reviewing new transcripts. Subsequently all transcripts were reviewed again and coded using the final dictionary of codes. The code words covered a wide range of topics including factors related to triggers of smoking, the woman's and others' reactions to her smoking during pregnancy, plan for smoking during pregnancy and after birth, influential roles of family and partner in woman's smoking or ability to abstain, and the woman's reaction to motherhood and her pregnancy.

Code words from each review were entered into ETHNOGRAPH and used for data-retrieval for thematic analysis. Additionally, each reader/reviewer developed a complete summary of each transcript, noting particular paradigms frequently discussed throughout the interview(s). The research team utilized two different methods of analysis for each transcript. First, frequency distributions of each code word were examined for issues more relevant to this group of women during their pregnancies. Secondly, specific contextual analyses were conducted to identify other paradigms related to each woman's smoking. For this purpose, we also aligned women's reports with those of their male partner's reports when available. Thus, we considered the women's perceptions of their smoking independently but also within the context of their intimate relationship with their male partners if their partner had provided information for this study.

Quantitative measures were entered, and frequency determinations made, to enable some description of characteristics of the study population. Text data and quantitative scores were linked so that some description of individual subjects was possible. Linkages between qualitative and quantitative information also described a context surrounding participants' reports to the open-ended questions.

## Results

### General description of study population

As depicted in Table 1, the mean age of the 50 subjects (37 women and 13 male partners) was 20.25 years for the women and 25.05 years for the male partners. The majority of women (84%) reported that they had obtained a high school diploma; 14% had attended only some high school; and the remaining 2% of women had attended college. Of the men, 85% had completed high school; 2 (15%) men had attended college. Forty-three percent of the women recruited for the study were single and not living with their partners; 27% of women cohabited with their significant other; and 19% were married. Regardless of their marital status, most women (97%) reported that their partner was currently involved in the pregnancy.

**Table 1:** Demographic characteristics and behavioral measures of female smokers and male partners

	Females (N = 37)		Males (N = 13)	
*Demographic variables*	Range	mean (SD)	range	mean (SD)

Age	17–25	20.25 (2.91)	19–44	25.05 (7.22)
Age onset of smoking	9–21	14.6 (1.97)	--	--
Duration of smoking (years)	0.5–15	5.3 (4.28)	--	--
FNTS	0 – 8	2.5 (1.99)	0–8	4.6 (2.10)
*Education completed (year)*	N (%)		N (%)	
Elementary	0 (00%)		0 (00%)	
Junior high/middle school	5 (13%)		0 (00%)	
High school	31 (84%)		11 (85%)	
College	1 (03%)		2 (15%)	
*Marital Status*				
Single	16 (43%)		---	
Married	7 (19%)		---	
Divorced	3 (08%)		---	
Living with significant other	10 (27%)		---	
*Partner involved in pregnancy*				
Yes	36 (97%)		---	
No	1 (03%)		---	
*Quantity of smoking (average #/day)*				
0–9	23 (62%)		2 (15%)	
10–11	6 (16%)		3 (23%)	
>12	8 (22%)		5 (38%)	
Missing			3 (24%)	
*Change of quantity*				
No smoking in the past week	10 (27%)		---	
Reduced quantity	17 (46%)		---	
Same quantity or increased	9 (24%)		---	
*FNTS score (Nicotine Tolerance)*				
0–5 (not dependent)	30 (81%)		5 (38%)	
6–11 (dependent)	4 (19%)		5 (38%)	
Missing			3 (24%)	
*BDI-II score*				
0–13 (minimal)	26 (70%)		10 (77%)	
14–19 (mild)	5 (17%)		2 (15%)	
20–28 (moderate)	3 (08%)		1 (08%)	
>29 (severe)	3 (05%)		0 (00%)	
*NLF score (authority)*				
5–11 (accept)	14 (38%)		3 (23%)	
12–18 (neither)	17 (46%)		8 (62%)	
19–25 (reject)	5 (14%)		1 (08%)	

Among the women, 26 (70%) were still smoking at the time of the interview, although 17 (46%) of these reported having decreased. Among the men, 9 (69%) were smokers; 1 (11%) of these men had ceased smoking. The mean age of smoking onset for the women was 14.6 years. Of those women who were still smoking, 54% reported that smoking before pregnancy was acceptable while 11% of the women expressed acceptance of maternal smoking after the birth of a child. Eleven percent of the men who were smoking reported that smoking after the birth of a child was acceptable.

Maternal depressive symptoms (BDI scores) ranged from 0 to 39; 6 (17%) of the women were experiencing moderate or severe depressive symptoms which corresponded to depressive symptoms being reported by only 1 (8%) male partner. Only three participants (15% of the women and 0% of the men) perceived themselves to have little control over events relating to parenthood. Fourteen percent of the women and 8% of the men highly rejected authority principals.

Proportionately more partners (38%) than the women (19%) who participated in this study endorsed a dependency on nicotine. The majority of women in this sample (81%) endorsed particularly low levels of nicotine dependence. Slightly less than half (46%) of the women reported ambivalence toward authority while 14% rejected the perceptions of authority figures. Sixty-two percent of the partners were ambivalent toward authority and only 8% completely rejected authority notions.

### Intra-personal role for smoking

Variables such as women's smoking status, depressive symptoms, and views of authority (Table 2) were examined by aligning individual's reports on the quantitative measures with qualitative reports found in the interviews and individual characteristics. These analyses revealed that beyond its social dimensions, smoking also assumed an important role in the intra-personal domain of the respondents; recurrent themes were those of 'stress" and "control".

**Table 2:** Individual profiles of women and male partners

Participant name	Age	BDI-II	NLF	FNTS
Ann	21	4	11	4
Kenneth (Partner)	25	4	14	5
Polly	17	11	11	--
Howard (Partner)	21	4	-	8
Gloria	20	1	12	3
Loretta	19	5	10	4
Christina	19	0	8	1
Amanda	20	7	8	--
Kera	20	1	13	4
John (Partner)	22	8	9	4
Jennifer	19	15	21	2
Sam (Partner)	27	0	19	4
Grace	25	-	--	--
Abbie	18	5	8	8
Jessica	23	10	19	1
John (Partner)	20	3	14	--
Khristi	20	-	-	--
Rosanna	18	14	16	0
Tiffany	23	6	13	4
David	26	-	-	--
Camilla	25	45	11	3
Ebony	18	18	19	1
Kimberly	18	0	17	--
Jocelyn	22	11	7	1
Tina	22	-	-	--
Jane	21	10	22	4
Mary	21	8	17	5
Denise	23	-	-	--
Samantha	26	-	-	--
Mike (Keisha's Partner)	25	17	9	2

Note. BDI score: 0–13 (minimal); 14–19 (mild); 20–28 (moderate); >29 (severe). NLF score: 5–11 (accept);12–18 (neither); 19–25 (reject).

Christina, a 19 year-old who continues to smoke but has thought about quitting stated:

"I sit here and often I think if I smoke a cigarette I will be okay...I get mad, but I don't even remember what I get mad over, but I smoke a cigarette..I'll deal with the problem.. [through]...smoking a cigarette".

Amanda [a current smoker] also sees a link between stress over situations which she cannot control and the need for tobacco:

"[I smoke] either when I have a really bad mood swing or I am nervous because like my boyfriend has not really been home from work yet, and it is like snowing."

The women's partners are aware of the role that stress plays in triggering cigarette use among the women, as suggested by Kera's [current smoker] partner, John [current smoker]:

"I think if maybe she could find a way to control some of the stress in her life a little better, that [the need to smoke] would not be a problem."

As articulated by Jennifer's [smoker] partner [smoker], men also employ tobacco to control stress:

"Say you are down on luck and you don't have it ... and your payday is not for two days and you are without cigarettes for two days...and that is running through your mind...And you hear kids screaming and people fighting and things like that and it gets to you and it is like you want that cigarette to calm everything down..."

However, despite the frequency with which respondents appears to respond to stress by smoking, some women, such as Mary [smoker who has recently quit smoking], suggested that this response might not achieve its goal:

"Whenever I am stressed, it feels like [smoking] helps, but it really doesn't. It is just sort of me".

For some women, the experience of pregnancy creates a heightened sense of stress and anxiety while others do not perceive changes in their sense of well being and lifestyle. The coping styles among those women who do perceive more stress and anxiety during pregnancy can vary. Women may look towards smoking to calm a sense of stress or anxiety. Abbie, an 18 year-old episodic smoker, noted that many of the daily stressors currently found in her life create a need for a cigarette:

"When I'm worried about the pregnancy or my next doctor's appointment, or Donald [non-smoker] is yelling at me because he doesn't understand my mood swings, I take a smoke and then I'm able to concentrate on that and calm down. And to think about something different."

Others may engage in alternative activities such as exercising or watching television to relax. Jessica, a 23-year-old smoker reports that,

"Now that I have tried to cut down on smoking, I've tried to do other things when I get stressed out. Sometimes I, you know, listen to my favorite music or watch shows on T.V. Most of the time it calms me down."

But, while smoking appears to serve as a means of *curbing *a heightened emotional state (i.e., stress) for many women, it also appears to be a response to a lowered emotional state (i.e., boredom). For example, Khristi [smoker who recently quit] noted that,

"Every time I was sitting and not doing something, I would light up a cigarette."

Christina [a smoker for many years] agreed with this paradox view of smoking and also described smoking more specifically as a "time filler" when she is bored,

"And my reason [for smoking] is pretty much because I am bored. So it's more of something to do. A filler...I smoked a lot...because I was stressed, but also because I was bored."

Other women move further to suggest that the behavior of smoking a cigarette rather than the need for a cigarette is the most important issue. Rosanna has been smoking for several years and argues that " [Smoking] just gives me something to do. It's not that I need it." Tiffany [has tried to reduce smoking] also distinguished between the behavior and the need,

"For me, I think, the act [of smoking rather than the need] is most important because as far as the smoking goes, I do not need it. I can quit it and not have the desire for it as long as I am busy doing something."

Men were aware of their partners' needs to keep occupied by smoking a cigarette. Kera's partner, John [a non-smoker] stated, "I think that she is one of those people that needs to be doing something and it's something for her to do...It's more the action of it rather than the smoking." Again, the distinction between the behavior of smoking and the need was made by many partners, often discounting the potential importance of any pharmacologic need, or dependence on, nicotine in the women's smoking behavior.

Partners also demonstrated a similar finding in their own smoking behaviors, especially once they had attempted to stop smoking for their partner's pregnancy. David, a 26-year-old, who had quit smoking stated:

"You definitely have to find something to do when you're not smoking, and it's amazing how much you don't realize how much time you spend smoking and then when you quit you're like "Wow! What have I been doing all the time?" You know I can sit here and twiddle my thumbs and before I'd be smoking a cigarette. So definitely it fills your time and gives you something to do...you miss the tactile feeling, and having the pack, buying the pack, opening the pack, getting a lighter..."

The perception of the role of smoking as an activity to engage in when one becomes bored did not appear to stop with the introspections of the women and their partners. Instead, many women perceived that boredom played a significant role in their friends' smoking behaviors. Howard [a smoker], reported that,

"Most of my friends are lazy...I mean they go and do their job and they just come home and sit because they have nothing else to do...Boredom is the reason why I think a lot of people smoke."

### Depressive symptoms and smoking

Factors such as the women's moods and their perceptions of others' reactions to their pregnancy and smoking also appear to be related to their smoking behaviors, attempts to quit smoking, and perceptions of smoking. For some women, increasing depressive symptoms appeared to be related to the women's negative perceptions of their pregnancies and their overall support systems. Camilla [a smoker who recently stopped] scored within the severely depressed range of the BDI-II, reported:

"I just want to die.' It was unreal.' [When I found out] I counted 200 times thinking about everything, like oh my God, what am I going to tell my dad, what am I going to tell everyone?' I'm not that excited about this [pregnancy]."

Camilla also thought that her partner and her family offered little support during this time:

"[My partner] and family do not want me to have the baby. They want to do their own thing."

Increased stress and worries since the beginning of pregnancy were also felt and verbalized by Rosanna, an 18 year old smoker who reported moderate depressive symptoms:

"There are many things going on now that I have a hard time dealing with. It gets to be a lot at times."

Like Camilla, Rosanna also perceives her social support network to be less than desired:

"I don't have many people to do things with. Not that I have the energy to do anything, but it would be nice to have the chance."

Women who reported greater depressive symptoms (as indicated by their BDI-II responses) commonly reported receiving no reward from smoking cigarettes, even though most of these women had not decreased the number of cigarettes they smoked each week. Camilla, who we heard from earlier about her lack of support stated,

"It [a cigarette] didn't really make me feel anything. It didn't help the situation any."

In contrast, some women who had fewer depressive symptoms were satisfied with their support and pregnancies. Jessica, a smoker who had a lower BDI-II score (Table 2), feels supported by her friends and partner and acknowledges the calming effect of smoking:

"My friends kind of look after me and Jon would do anything to help me... [Smoking] helps me calm down and enjoy things a little more."

By their reports, Jessica seems to get more rewards from smoking than Camilla and is more satisfied with the support she receives. Differences in these women's perceptions of support and satisfaction from smoking may be due to their socio-emotional states (e.g., depressive symptoms).

### Views of others and smoking

Other factors such as an individual's views and rebelliousness towards authority may also have an impact on her perceptions of smoking and the pregnancy, desire to quit, and sense of support. A woman's rejection of authority may influence how she begins to smoke and whether she quits. For many women, family members, close friends, and a partner may be perceived as authority figures. Jessica's score on the Naysaying Low F scale indicated rejection of authority. Her views of authority were reflected in her view of smoking during her pregnancy:

"It started as part of an image that you have of yourself and after a while you begin to feel tougher [when you smoke]."

Jessica's perceptions of authority also appear to infiltrate her perceptions of smoking while being pregnant:

"I am kind of scared, if somebody tried to say something, it would upset me, it would upset me a lot.' I have become very attached to this kid, and I do not want anyone to mess with me about it."

Jessica also thinks about how others judge her when spending time with friends who smoke but appears to become defensive while talking about changing her behavior:

"I worry about what people are going to think about what kind of mother I am when I take my baby [to work] but I am not going to give up my friends [who smoke], I am sorry but I am not."

Other women who reject authority principals also discuss a similar frustration toward the way others appear to judge them. Ebony, an 18-year-old smoker stated:

"It is very hard to quit.' I would not tell anybody to quit because I am a smoker too and that is an invasion."

### Social role of smoking

Smoking assumed a prominent role in the social relations of many of the women – both in their relationships with their partners, their wider sphere of friends, and their family. The nature of the roles played by smoking in their relationships was dynamic and seemed to have changed considerably since the pregnancy began.

Many women reported that smoking was a shared activity – both with their partner and with their friends. For some of these women, the loss of this activity since the pregnancy seemed to represent the loss of an opportunity for intimate sharing. For example, Ann, a 22-year-old woman who recently quit smoking, reports that she and her partner spend less time together now that she has stopped smoking:

.....because he is smoking most of the time. And I guess we spent more time together when we both smoked, especially when we were at my house because we were not allowed to smoke at my house, only in a certain area, so we had to leave together any time we wanted to smoke....Whenever we both smoked we both shared it, either I would light one and he would smoke half or he would light one, smoke half, and give it to me. Just throughout the day we would just smoke like that.

Her sentiments are echoed by the 21 year old partner of Polly, who continues to smoke up to nine cigarettes a day. He observed that:

I just got out of a relationship with someone who did not like smoking and I cannot have that. You know I cannot be around someone who doesn't want me doing something like that. But mainly that is why I thought we fit because we both smoked and I did not have to worry about hearing it from someone else to quit. Because when you tell me to quit more, I do not want to.

The bond provided by smoking, with its corresponding loss as a result of smoking cessation, appears not to be limited to intimate relationships. Ann again noted: "Basically I would smoke if I was out with my friends...Uh, some of the friends that I used to go smoke with you know I don't really go talk to them as much, the people that I used to go outside and smoke with. So, I miss that part..."

Ann's partner, Kenneth, recognized the role of smoking in her life:

"Social, meaning it is a bonding experience or it is easier to talk whenever she smokes ... and she would get to her friends' house and she will visit there and kill a couple of cigarettes in a couple of hours."

Likewise, Gloria, a 20 year-old who recently quit smoking, commented,

"I feel left out of some situations with my friends. I guess it's because they like go do things without me because they know I can't be around them while they smoke..."

Loretta, a 19 year old who is thinking about quitting recognizes the impact that her decision may have on her friendships:

"Yeah, all my friends smoke. And if I quit I don't want none of them smoking around me. One thing that is going to be a hard thing, you know, you can't come to my house."

### Smoking and emotional discord

The change in status with regard to the role of smoking between partners and friends is a frequently expressed source of tension. Among couples with discordant smoking status, almost uniformly the woman has decreased or stopped smoking without an equivalent change in her partner, the discordance is a prominent theme in conversations. Ann observed that:

I think [quitting] has been really bad. It makes me angry that he is still smoking and I have quit. So it has an impact ...in some ways and it makes us argue a little more when he smokes....He says that 'you had to quit and I should not have to be forced to quit also.' And he thinks I am being rude to him ...because if we are in the car, I will not let him smoke...

Likewise, Kimberly declared that,

".... it just irritates me that he tells me to stop smoking but he has to be a part of it too. And I just get angry because I know that the baby is not in his belly but it just irritates me that he is telling me to stop but he does not stop..."

Jocelyn is a 22-year-old who continues to smoke up to nine cigarettes a day. Although smoking has been a source of tension between her and her partner, she places this discord in a broader context, vacillating between anxiety that he has not yet begun to assume the role of parent, but ending with an expressed confidence that he will eventually do so.

"I know that it is different for me than him but I don't really find it much different because I felt like if he is going to criticize me and tell me to stop smoking then he should stop smoking. But then again, he is not the one carrying the baby. And I just don't think he will stop. Like sometimes I will want him to stop smoking 'cause whenever I have the baby, there will be no smoking inside the house. Sometimes I get mad but not as much as I used to before.....I am thinking (the baby) might make us closer because I think it might change his life...(the) baby will make him settle down more because he is still in the role of going out and partying. I think that it might make us closer because he still likes to get out and hang out with his friends you know. And I think the baby will be the most important thing and I think he will be attached."

Jocelyn also recognizes that her discontinuation of smoking reflects a more global movement away from a former lifestyle which she used to share with her friends-but again her optimism emerges, this time with the conviction that her friendships will endure despite the strain placed by changing lifestyles:

"...like my friends, like me, I was into partying a lot like hanging out at the bar drinking, that is how my friends are still now...but I am not into that because I don't think that it is a safe environment for me to be in. And they understand our relationship has changed. We will still do things..."

The partners of the women in the study also appear to recognize the tension that discordant smoking status breeds in a relationship. As Tina's partner explained:

"I stopped for almost a month...But she was still smoking and then I started smoking again. It seemed like because I wasn't smoking she had a low tolerance for me, and she like, you know, we got into arguments about it..."

He elaborated that his partner was aware that likewise, if she were to discontinue smoking (now that he was smoking again) their relationship would be negatively impacted:

"Well, she tells me that it is too hard for her not to smoke, she says that if she stops smoking she believes that she will be ---- like I wouldn't want to be around her, in other words.... She would be cranky..."

Jane has decreased the number of cigarettes she smokes each day since learning of her pregnancy and reports being physically repelled by cigarette smoke. However, she does not attribute this revulsion to her pregnancy, but rather to her decreased smoking status:

Since I haven't smoked so much, the smell of my boyfriend, I mean, it's enough to turn my stomach...I can tell if I give him a kiss and it's on his breath and even under all that grease and sweat...cause he is a cook.... You smell that and the smoke on top of it. And his teeth are real bad. After a while you just get tired."

Her partner shares her perceptions both that her tolerance is lower and that this reduction results from her own abstinence:

"If she doesn't smoke then she will be like, well that stuff is disgusting. It makes me wanna puke."

The reports of these women and their partners illustrate that discord between partners appears to be a prominent concern for individuals (women or their partners) whose attempts to quit smoking are not matched by their partners' own efforts to quit. Conflict appears to be minimized if both partners attempt to quit smoking together. However, not all couples with incompatible smoking interests appear to engage in conflict. In particular, women who feel supported by their partners in their efforts to refrain from smoking, as well as their pregnancy experiences in general, do not appear to resent the imbalance or view it as an unshared burden. Jessica relates the story about her partner (non-smoker) that,

"I asked him to buy cigarettes for me once because I was really losing my mind and he would not do it. And he seemed very supportive."

When asked how she would describe someone who was "supportive" she responded:

"Try to figure out just exactly what it is I need to hear, somebody that I can trust to tell me the truth."

She elaborated that her partner:

...Would tell you that his first responsibility is to take care of me and the baby and then take care of school and I would tell you that his first responsibility was to take care of himself and then school and I will worry about me and the baby...but he is a good guy.... He is glad that I quit. It makes it a little harder on him though because I used to get upset and go smoke a cigarette and now I get upset and...end up having a fight with John...

Jessica appears to feel supported and appreciated for her efforts. By contrast, Mary, who is attempting to remain abstinent, expresses anger that her partner both continues to smoke and does not praise her for her efforts:

"He smokes too and he never says anything. He should be like 'you're doing real good. You should be real proud of yourself. You quit smoking.' Don't hear it from nobody else, so I guess I gotta do it myself."

Like the absence of praise, women complained about "support" efforts such as those offered by one partner: "I just harp on her about cutting back". Similarly, Denise does not find her partner's (non-smoker) approach helpful: "He said "I am not smoking and I'm not paying for it.' Now he's on my case to quit smoking."

### Smoking cessation: the cognitive perception of cessation as a burden or a gift?

Given the importance of smoking in social interactions and intra-personal coping, it might be expected that smoking cessation would present a formidable burden and/or represent a significant loss to women, particularly during the prenatal period when their social and biological spheres may be changing significantly. Indeed, some women view smoking cessation as a major imposition or burden. However, others appear to regard it as gift that they can offer to positively affect their child's health.

Samantha reported that she stopped smoking when she learned that she was pregnant:

"It makes me feel better. Sometimes it makes me feel better about myself. You know, that I can do something."

Keisha's partner, who also stopped smoking upon learning of her pregnancy related his thoughts at the time: "A positive thing would be that I quit smoking and I can teach my kid that now."

By contrast, other women are unable to change their smoking behaviors following the notification of their pregnancies. Kimberly, for example, has not been able to make this commitment. She reflects, "I need to smoke because there is a lot of stress that I am dealing with right now with like the father and just me giving up everything that I used to have fun..." Indeed, the general theme of self-denial imposed by pregnancy and childbearing is interspersed throughout the dialogue of many of those still struggling with tobacco. Mary reports isolation from her partner both as a result of decreased smoking and of the pregnancy itself:

We both used to smoke like a pack a day. Now he smokes maybe a half a pack but he is never around me...We are not as cuddly as we used to be. We don't talk about each other as much; it's always the baby, the baby. I think we have grown apart in the aspect that we don't talk as much as we used to, I mean we will stay up talking, but it will be about the baby.

Her reports may describe a loss of social context that once was filled with mutual smoking behaviors and meaningful discussion. However, she describes their relationship as having changed both physically in terms of the smoking but also in their intimacy and discussions. In this circumstance, the social context surrounding various relationship and social behaviors (e.g., smoking) have gone through a transformation during her pregnancy.

In contrast, Jessica, who we heard from earlier as she described feeling supported by her partner (non-smoker) in her successful efforts to quit, also recognizes, but embraces, the sacrifices she will make as a mother:

I expect (mothering) to be difficult. I expect it to consume all my time most of my energy and a fair portion of my sanity. It is going to be fun too, but there is this little person that depends on me for everything and has no way to get across to me what she wants other than scream.... To an extent, yeah, I am going to lose part of my social life and I am not going to be able to hang out with my friends every time I feel like it --- and I am hoping our friendships will still survive.

## Discussion

These interviews of young women and their partners early in their pregnancies reveal cigarette smoking to be more than an isolated addictive activity. We see instead a behavior woven into the social and personal terrain of a world being transformed by a profound awareness of changing roles and new accountabilities. Design of effective means to assist pregnant women in smoking cessation may require accommodation and sensitivity to key features of this unique and shifting landscape, including those we have highlighted here: for all, awareness of responsibility to the infant, and a new context for relationship to partner and friends, and for many, depressive symptoms, stress and boredom.

All of our subjects were aware of the risks of smoking in pregnancy, but the extent to which the challenge of quitting was viewed as an undesirable burden, as opposed to a welcome opportunity to serve the interests of their anticipated newborn varied widely. A sense of self-efficacy, and confidence in ability to parent may determine how emerging responsibilities and sacrifices are perceived and addressed [9,34-36]. To the extent that smoking and its associated meanings are integrated into self-perception, abandonment of smoking may aggravate the challenge of adopting a parental self-concept for those women who view their new role with resentment or fear.

Change in smoking behavior in relation to intimate and social relations was a feature, if not a focal point in the drama of adoption of a new self. Our subjects uniformly acknowledged an impact of impending motherhood on their relationships. Often, for these young smokers, the need to stop smoking and avoid smoke is seen as a complicating dimension of this adjustment. We observed that sometimes conflict was increased when our pregnant subjects were attempting to quit and their partners continued to smoke, as has been reported elsewhere [36-38]. In some instances this dissonance affected the woman's perception of her partner's ability or willingness to share in the emerging responsibilities of parenthood. Further, an impact of the obligation to change smoking behavior on patterns of social activity was consistently reported. The importance of smoking in the social realm has been observed in other settings, and social support is a known aid to quitting [39-42]. The alteration of associated social patterns that accompanies smoking cessation has been reported and usually incurs a sense of loss as was often the case among the women we interviewed [42].

The low number of women with depressive symptoms is not what one would expect given the cultural expectation that pregnancy be a time of happy anticipation. For these young women especially, quitting competes with the sense of self-reward offered by smoking, and nicotine dependence itself may enter into the cycle of rumination and absence of a sense of self-worth [43]. Interestingly, women who were experiencing greater depressive symptoms seemed less likely to describe smoking as a satisfying experience. Some women who reported greater depressive symptoms were able to quit smoking during their pregnancies. These findings could point to a hierarchical shift in women's belief system, particularly in terms of the perceived importance of smoking when compared to the adjustment of impending parenthood or within the realm of depressive thoughts. Alternatively, we must consider reasons why some women who had reported having depressive symptoms and an apathy toward smoking had not quit smoking. Can individuals develop a strong lack of motivation to change their behavior even in the absence of interest in the behavior? What factors lead to this contradictory behavior to continue to engage in a behavior that is no longer reinforcing? Perhaps their smoking behavior continued to be reinforcing but the way in which this occurred had changed during this period of time.

Views of the perceptions of authority figures regarding one's smoking behavior during pregnancy also appeared to be a presenting factor with regards to the woman's decision to quit smoking. However, these views of authority may act indirectly by determining the woman's reaction to others' views of her. For example, if she rejects others' views she may react by smoking publicly or increasing the number of cigarettes she smokes. If she accepts others' views and opinions, she may be more likely to quit smoking as a result of a perceived public rejection of her smoking behavior.

The goal of this research was to gain insight into the personal meaning of smoking for young pregnant women that would yield avenues for more effective interventions to reduce tobacco use. Previous researchers such as Reid [44] have demonstrated and argued that the current "stages of change"-based approaches are not suitable for individuals who are experiencing changes in their development (e.g., adolescents). Indeed, our preliminary findings suggest that the milestone changes associated with pregnancy create a chaotic environment where the implementation of a changed-based intervention is not appropriate and therefore, not effective. To date, best practice behavioral interventions to reduce smoking among pregnant women have yielded disappointing rates of success. From this limited sample of newly pregnant women, objectives for further inquiry across the span of pregnancy and among a larger sample of women are revealed; the themes of adaptation to a new self, relations with partners and friends, and coping with stress are prominent in our sample, however further examination of the societal and cultural influences is needed. Strategies for smoking cessation in pregnancy that acknowledge and address or, perhaps capitalize on these themes may provide much improved outcomes for women and their infants. These findings could help to target intervention efforts, a strategy that may be important for future public health initiatives.

Current cessation programs among pregnant women are not as successful as once hoped. We hypothesize that programs which focus on the multiple transitions a woman faces during this period in her life (e.g., parenthood, changing role in relationship, and change in smoking status) would be more successful in accommodating her needs and thereby reducing her physiological or psychological urge to continue smoking. These findings may identify potential issues to consider and attend to with a pregnant woman who is trying to quit smoking but is having difficulties. Authors may with to identify and evaluate empirically based intervention methods for pregnant women identifying specific components essential to improving their overall quit rates.
